# A Rare Case of Spontaneous Pneumothorax Leading to Cerebral Air Embolism

**DOI:** 10.7759/cureus.52277

**Published:** 2024-01-14

**Authors:** Zakaria Alagha, Amro Al-Astal

**Affiliations:** 1 Internal Medicine, Marshall University Joan C. Edwards School of Medicine, Huntington, USA; 2 Internal Medicine/Pulmonology, Marshall University Joan C. Edwards School of Medicine, Huntington, USA

**Keywords:** patent foramen ovale (pfo), usual interstitial pneumonia (uip), transient ischemic attack, paradoxical cerebral embolism, cerebral air emboli, pneumothorax ptx

## Abstract

Cerebral arterial air embolism (CAE), a rare subtype of air embolism, carries a 21% mortality rate. We present a unique case involving a 69-year-old female with a history of usual interstitial pneumonia (UIP) who suffered a transient ischemic attack (TIA) due to CAE. Unlike typical cases, CAE in this instance resulted from spontaneous pneumothorax, not the more common iatrogenic causes. Adding complexity, an unexpected discovery emerged during evaluation: a patent foramen ovale, contributing to paradoxical embolism. This underscores the vital need to consider CAE as a differential diagnosis in UIP patients with neurological symptoms, highlighting its rarity and diagnostic challenges.

## Introduction

Air embolism (AE) typically arises as an iatrogenic complication secondary to invasive procedures, with cerebral arterial air embolism (CAE) being a rare and severe subtype associated with high mortality rates [1]. In this report, we present a unique case where a 69-year-old female presented with a transient ischemic attack (TIA) resulting from CAE secondary to spontaneous pneumothorax, which is distinct from documented iatrogenic CAE cases. The case is further complicated by the presence of a previously undiagnosed patent foramen ovale (PFO), highlighting the exceptional rarity and clinical challenges associated with such occurrences.

## Case presentation

**Figure 1:**
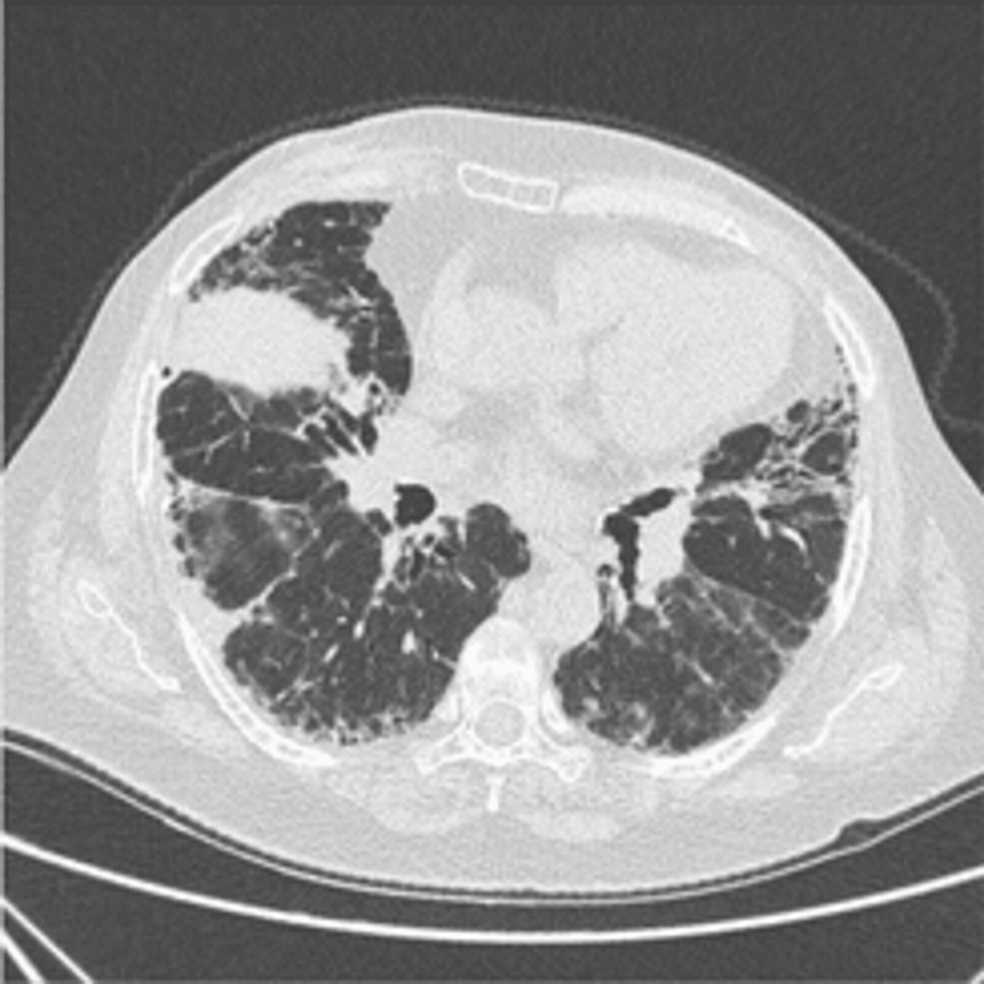
High-resolution computed tomography (CT) scan of the chest showing basilar predominant coarse interlobular septal thickening with peripheral bronchiolectasis. Early subpleural is honeycombing within both lung bases.

A 69-year-old female patient, with a history of usual interstitial pneumonia (UIP) (Figure 1), experienced a sudden episode of expressive aphasia and right-hand weakness lasting five minutes, resembling a TIA.

The patient maintained stable vital signs throughout the hospital stay. The physical examination revealed diminished breath sounds over the right upper lung, but was otherwise unremarkable, notably lacking any signs of lower extremity swelling indicative of deep vein thrombosis (DVT). No atrial fibrillation was recorded during the telemetry monitoring. Laboratory results were also unremarkable. The bilateral lower extremity venous Doppler was negative for DVT.

**Figure 2:**
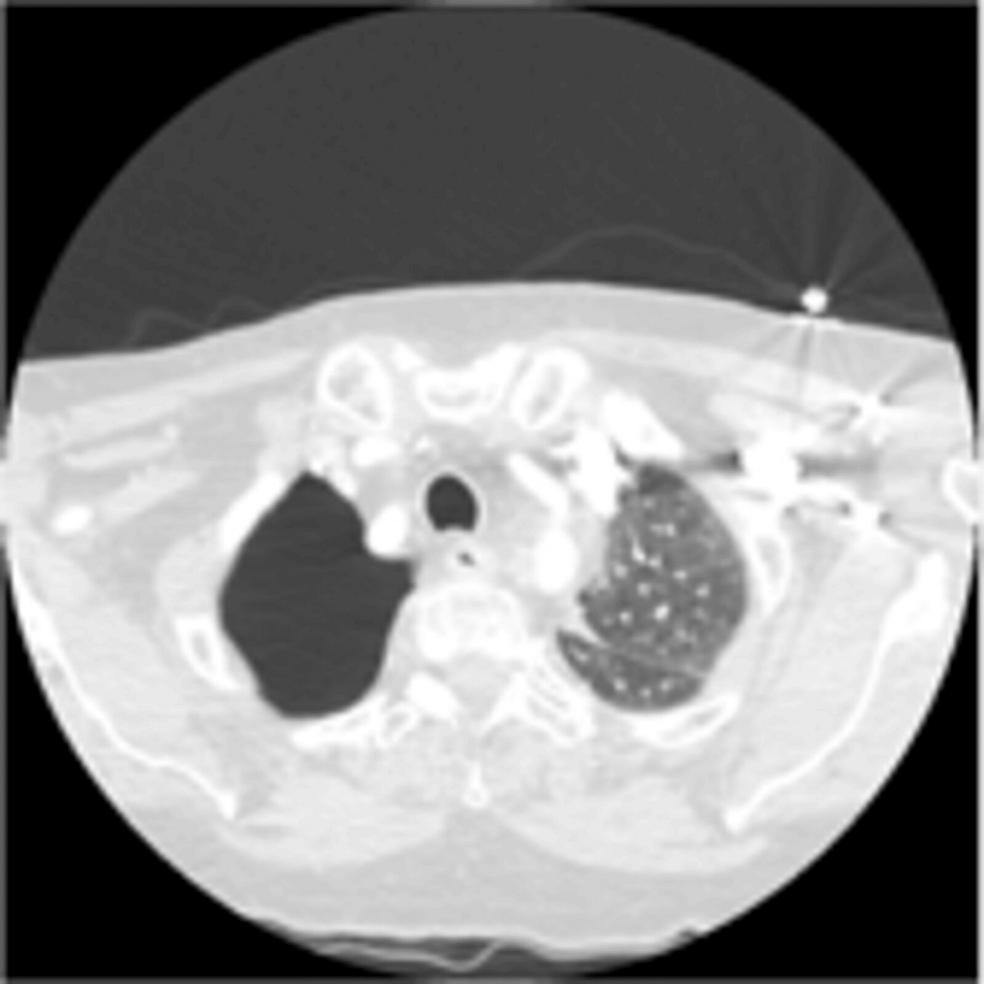
Lower cuts of the computed tomography angiography(CTA) scan of the neck showing a right apical pneumothorax

A CT angiography (CTA) scan of the head and neck, conducted during the TIA evaluation, showed no neurological pathology, and both vertebral and carotid arteries appeared widely patent. Unexpectedly, the scan revealed a significant right-sided pneumothorax in the lower cuts of the CTA neck (Figure 2).

Echocardiogram, as a part of the TIA workup, yielded a surprising result: a PFO. This discovery raised the possibility of a paradoxical embolism causing the initial TIA-like symptoms. While conservatively managing the TIA, prompt chest tube placement addressed the pneumothorax. However, an ongoing air leak complicated the clinical course. Successful video-assisted thoracoscopic surgery (VATS) resolved the pneumothorax. Remarkably, against all odds, the patient was discharged home in improved health.

## Discussion

The clinical presentation of patients with air embolism often exhibits atypical symptoms, posing a challenge for accurate diagnosis. The incidence of iatrogenic air embolism is reported at a rate of 2.65/100,000 admitted patients. The implications of an air embolism are contingent upon several factors, including the volume and velocity of the air, the route through which air enters the bloodstream, and the specific location at which it enters the blood vessels. In cases where air emboli traverse the systemic circulation, they may gain access to the left heart artery system by way of the septal defects or PFO, potentially culminating in embolisms affecting the brain, extremities, and visceral organs - a phenomenon known as paradoxical embolism. Notably, a substantial 77% of air emboli find their way into the left heart artery system through the PFO or septal defect. When air emboli enter the cerebral arteries, they can precipitate grave consequences. The fatality rate associated with CAE can be as high as 21% [1-3].

Air embolism can also result from emboli entering the right heart system due to elevated pulmonary artery pressure, reducing pulmonary blood flow, lowering left heart volume, and potentially opening the foramen ovale due to left-right atrial pressure differences. Abnormal connections between the right and left heart, like septal defects and nonoccluded arterial ducts, can similarly contribute to paradoxical embolisms. Moreover, air embolism can trigger the release of inflammatory factors from endothelial cells, exacerbating secondary vasospasm and capillary leakage [3].

CAE is a relatively uncommon clinical condition characterized by symptoms such as focal neurologic deficits, coma, seizures, encephalopathy, and headache. Typically, CAE arises as an iatrogenic complication, often occurring in conjunction with central venous catheter placement or removal, various endoscopic procedures, or as a result of trauma or surgical interventions [1,4].

Pneumothorax, an uncommon factor that contributes to CAE, particularly in cases of secondary spontaneous pneumothorax (SSP), is associated with elevated morbidity and mortality, with an annual incidence of 6.3 and 2 cases per 100,000 in men and women, respectively. Most patients with SSP exhibit compromised lung function due to underlying conditions, making it a potentially life-threatening condition necessitating prompt intervention. The risk of adverse outcomes from pneumothorax is worsened with lower baseline forced vital capacity (FVC) levels. Interestingly, despite the severe restrictive pattern in our patient's baseline lung function (FVC measuring 37% of predicted value), she has responded well to treatment [5].

In cases of pneumothorax where intrapleural pressure increases, the potential for air molecules to enter bronchial veins due to pressure gradients is a concern. When a patient with an undiagnosed PFO is involved, this becomes particularly worrisome, as it can lead to paradoxical embolism. In our patient's case, the previously undiagnosed PFO likely played a pivotal role in this paradoxical air embolism secondary to spontaneous pneumothorax [3].

When it comes to CAE, findings from CT or MRI scans can provide supportive or confirmatory evidence for diagnosis. However, it's crucial to emphasize that normal imaging results cannot definitively rule out CAE. In a dog model, CT scans detected only 20% of CAE cases when 0.25 mL of air was injected, and it took 2 mL of injected air to achieve 100% sensitivity in detecting CAE. Furthermore, in various studies involving deep-sea divers experiencing neurological and pulmonary symptoms indicative of venous air embolism, no CT evidence of cerebral air embolism was observed. Consequently, clinical evaluation remains the preferred method for assessing CAE. In our patient, despite negative imaging findings for CAE, we postulated that CAE likely triggered TIA, as our patient has a Risk of Paradoxical Embolism (ROPE) score of 5, suggesting a 34% chance that the stroke is attributed to a PFO [6].

The treatment for CAE involves critical components. First, volume resuscitation is essential to counter hemoconcentration, which thickens the blood and disrupts microcirculation. This is achieved by restoring normal blood volume with colloid infusions, elevating venous pressure to prevent further air entry into veins. Second, ensuring adequate oxygenation is crucial by increasing the fraction of inspired oxygen to reduce the air embolus size and improve organ ischemia. While hyperbaric oxygen therapy is not the primary treatment for CAE, it may be considered for cases with significant neurological deficits, ideally initiated within four to six hours but beneficial up to 30 hours post-embolism. Notably, anticoagulation and corticosteroids are generally discouraged in CAE treatment [1,3,7].

The management of SSP poses significant challenges, with treatment options ranging from pleurodesis to chest tube insertion or VATS in severe cases. Chemical pleurodesis is a non-surgical option but may be challenging due to lung rigidity. Importantly, hospital mortality rates for IPF-related pneumothorax range from 4.5% to 17.9%, with a notable recurrence rate of 70.6% [8].

## Conclusions

This case aims to highlight the importance of considering CAE as a potential differential diagnosis in patients with UIP experiencing neurological symptoms and emphasizes the need to consider the possibility of paradoxical air embolism and the potential existence of a shunt in similar clinical scenarios.
